# The Influence of Vitamin D Receptor Genetic Variants on Bone Mineral Density and Osteoporosis in Chinese Postmenopausal Women

**DOI:** 10.1155/2015/760313

**Published:** 2015-02-17

**Authors:** Wei He, Ming Liu, Xiaonan Huang, Zuhong Qing, Wei Gao

**Affiliations:** ^1^Department of Orthopedics, The 305 Hospital of Chinese PLA, Beijing 100017, China; ^2^Department of Chemistry, Capital Normal University, Beijing 100037, China

## Abstract

Growing evidence indicates that the vitamin D receptor (*VDR*) gene is an important candidate gene for influencing the development of osteoporosis. The aim of the study was to evaluate the potential association between genetic variants of *VDR* gene and bone mineral density (BMD) and osteoporosis in Chinese postmenopausal women. The study included 970 Chinese postmenopausal women at the postmenopausal osteoporosis (482) and healthy controls (488). The BMD of lumbar spine (L_2–4_ anterior-posterior view), femoral neck hip, and total hip was evaluated using the Norland XR-46 dual energy X-ray absorptiometry (DEXA). The genotypes of *VDR* genetic variants were determined by the created restriction site-PCR (CRS-PCR) and confirmed by DNA sequencing methods. Our data indicated that the *VDR* p.Glicine (Gly)14 alanine (Ala) and p.histidine (His) 305 glutanine (Gln) genetic variants were statistically associated with adjusted femoral neck hip BMD, adjusted lumbar spine BMD, and adjusted total hip BMD (*P* values < 0.05). Results from this study suggest that the *VDR* p.Gly14Ala and p.His305Gln genetic variants are significantly associated with BMD decrease in Chinese postmenopausal women and might be used as molecular markers for assessing the risk of BMD and osteoporosis.

## 1. Introduction

Osteoporosis, an important and complex health concern in the postmenopausal women, is a polygenic and multifactorial disease that is characterized by the increased risk of fragility fractures and reduced bone mineral density (BMD) [1–11]. In the past decade, many published studies have reported the potential association of environmental and genetic variants with BMD and osteoporosis [8–33]. It is well accepted that the genetic factors play key functions in the pathogenesis of osteoporosis [34–39]. However, the exact etiology of osteoporosis still remains poorly understood. In recent years, the vitamin D receptor (*VDR*) gene has been considered as an important candidate gene in the modification and the development of BMD and osteoporosis [8–32]. The* VDR* gene contained 14 exons and located on chromosome 12q12–q14, which is a member of the nuclear receptor family of transcription factors [11, 40, 41].* VDR* modulation influences the expression and transcription of genes which involved in bone mass formation and calcium uptake, such as osteocalcin and calcium-binding proteins [11, 42]. The* VDR* gene is polygenetic gene, and single nucleotide polymorphisms (SNPs) of* VDR* gene could influence the expression and function of* VDR* protein, which are proved to influence the risk of BMD and osteoporosis. Morrison et al. firstly investigated that genetic variants in the* VDR* gene could predict spinal and femoral BMD in Caucasian women [12, 13]. Since then, a large number of epidemiologic studies have reported the* VDR* genetic variants (e.g.,* Fok*I (rs10735810),* Bsm*I (rs1544410), and* Apa*I (rs7975232)) are associated with BMD and osteoporosis in different ethnic groups [8–32]. However, to date, there are no published similar studies on the potential relationship of p.Glicine (Gly)14 alanine (Ala) and p.histidine (His) 305 glutanine (Gln) genetic variants in* VDR* gene with BMD and osteoporosis. Therefore, considering the important role of* VDR* gene in the pathogenesis of BMD and osteoporosis, the objective of this study was to investigate the p.Gly14Ala and p.His305Gln genetic variants of* VDR* gene and evaluate the potential association of these two SNPs with BMD and osteoporosis in Chinese postmenopausal women.

## 2. Subjects and Methods

### 2.1. Study Subjects

We enrolled 970 Chinese postmenopausal women containing 482 at the primary postmenopausal osteoporosis (aged 45–86 years) and 488 healthy controls (aged 43–89 years) in this case-control study. All subjects were recruited from the 305 Hospital of Chinese PLA between February 2010 and March 2014. All participants were genetically unrelated Chinese Han ethnic. Subjects with primary postmenopausal osteoporosis were diagnosed and confirmed by doctors. Individuals with a past or present history of diseases known to affect bone metabolism or those who were taking drugs which could affect bone metabolism were excluded from this study. The protocol of this study was approved by the local ethics committee of the 305 Hospital of Chinese PLA. Informed consent was obtained from all subjects enrolled in this case-control study.

### 2.2. Bone Mineral Density Measurement

The BMD of lumbar spine (L_2–4_ anterior-posterior view), femoral neck hip, and total hip was evaluated using the Norland XR-46 dual energy X-ray absorptiometry (DEXA) (Norland Coopersurgical Corp, WI, USA) [43]. The BMD values were automatically calculated from bone mineral content (g) and bone area (cm^2^) and expressed as g/cm^2^. All data are shown as mean ± SD (SD, standard definition of the mean, BMD values adjusted by age, height, and weight).

### 2.3. DNA Extraction and PCR Amplification

Peripheral venous blood was collected from each enrolled subject in this case-control study. Genomic DNA was isolated from blood using the QIAamp DNA Blood Mini Kit (QIAGEN, GmbH, Germany) and then stored at −80°C until analyzed. According to the human* VDR* gene's DNA sequences (GenBank ID: NG_008731.1) and mRNA sequences (GenBank ID: NM_000376.2), the specific primers of polymerase chain reaction (PCR) were designed using the Primer Premier 5.0 software (Premier Biosoft International, Palo Alto, CA).  Table 1 shows the detailed information of the primer sequences, region, annealing temperature, and fragment size. The PCR amplifications for genetic variants of* VDR* gene were performed in a 20-*μ*L reaction mixture, which containing 50 ng template DNA, 1x buffer (100 mmol Tris-HCl, pH 8.3; 500 mmol KCl), 0.25 *μ*mol primers, 2.0 mmol MgCl_2_, 0.25 mmol dNTPs, and 0.5 U Taq DNA polymerase (TaKaRa, Dalian, China). The PCR cycling protocol was carried out by an initial denaturation at 94°C for 5 min, followed by 32 cycles of 94°C for 30 s, annealing at the corresponding temperature (shown in Table 1) for 30 s, and 72°C for 30 s, with a final extension at 72°C for 10 min. The PCR products were separated on electrophoresis in a 2.0% agarose gel stained including 0.5 *μ*g/mL ethidium bromide and observed under ultraviolet light.

### 2.4. Genotyping

The genotypes of genetic variants p.Gly14Ala and p.His305Gln in* VDR* gene were determined by the created restriction site PCR (CRS-PCR) method with one of the primers containing a nucleotide mismatch, which enables the use of restriction enzymes for discriminating sequence variations [44–48]. Following manufacturer's instructions, the amplified PCR products (10 *μ*L) were digested with 2 U selected restriction enzymes (given in Table 1) at 37°C for 10 h. The digested PCR products were electrophoresed on a 2.5% agarose gel containing 0.5 *μ*g/mL ethidium bromide staining for 1 h at 100 V, and different genotypes of* VDR* genetic variants were visualized directly under UV light. In order to confirm the concordance of the genotyping results from CRS-PCR method, we selected random samples (20% of total samples) to be reanalyzed by the DNA sequencing method (ABI3730xl DNA Analyzer, Applied Biosystems, Foster City, CA).

### 2.5. Statistical Analyses

All statistical analyses were analyzed by the Statistical Package for Social Sciences software (SPSS, version 16.0; SPSS Inc.; Chicago, IL, USA). The chi-squared (*χ* ^2^) test was utilized to compare the distributions of allelic and genotypic frequencies in the studied subjects. The genotype frequencies of* VDR* genetic variants were tested for Hardy-Weinberg equilibrium (HWE). Multiple regression analyses were performed to evaluate the potential relationships between the variables. All data were expressed as mean ± SD. The significant level was set at *P* < 0.05.

## 3. Results

### 3.1. Identification and Genotyping of* VDR *Genetic Variants

Through the CRS-PCR and DNA sequencing methods, we detected two* VDR* genetic variants (p.Gly14Ala and p.His305Gln) in this study. Our DNA sequencing analyses suggest that the p.Gly14Ala is a nonsynonymous mutation, which caused G→C mutation in exon3 of* VDR* gene and resulted into Gly to Ala amino acid replacement (p.Gly14Ala, reference sequences, GenBank IDs: NG_008731.1, NM_000376.2, and NP_000367.1). The PCR products were digested with the* Mae*I restriction enzymes and divided into three genotypes, GG (195 and 18 bp), GC (213,195 and 18 bp), and CC (213 bp, Table 1). As for p.His305Gln, our DNA sequencing analyses indicate that it is also a nonsynonymous mutation, which caused C→G mutation in exon9 of* VDR* gene and resulted into His to Gln amino acid replacement (p.His305Gln, reference sequences, GenBank IDs: NG_008731.1, NM_000376.2, and NP_000367.1). The PCR products were digested with the* Mae*III restriction enzymes and divided into three genotypes, CC (189 and 17 bp), CG (206,189 and 17 bp), and GG (206 bp, Table 1).

### 3.2. Allelic and Genotypic Frequencies

The distributions of allelic and genotypic frequencies for p.Gly14Ala and p.His305Gln genetic variants of* VDR* gene are summarized in Table 2. The allele-G and genotype GG of p.Gly14Ala and allele-C and genotype CC of p.His305Gln genetic variants were predominant alleles and genotypes in the studied subjects, respectively (Table 2). As for p.Gly14Ala, we found significant differences between the allele frequencies of cases (G, 64.11%; C, 35.89%) and those of healthy controls (G, 69.67%; C, 30.33%, *χ* ^2^ = 6.7816, *P* = 0.0092). Besides, the genotypic frequencies in cases (GG, 43.57%, GC, 41.08%, CC, 15.35%) were statistically significant different from healthy controls (GG, 47.13%, GC, 45.08%, CC, 7.79%, *χ* ^2^ = 13.6018, *P* = 0.0011, Table 2). As for p.His305Gln, significant differences were detected between the allele frequencies of cases (C, 67.53%; G, 32.47%) and those of healthy controls (C, 72.34%; G, 27.66%, *χ* ^2^ = 5.326, *P* = 0.0210). Besides, the genotypic frequencies of cases (CC, 46.06%; CG, 42.94%, GG, 11.00%) were not consistent with those of healthy controls (CC, 54.30%; CG, 36.07%, GG, 9.63%, *χ* ^2^ = 6.6290, *P* = 0.0364, Table 3). The chi-squared (*χ* ^2^) test indicates that these two* VDR* genetic variants were fitted within HWE (all *P* values > 0.05).

### 3.3. Association between* VDR* Genetic Variants and Bone Mineral Density

The value of weight, age, height, body mass index (BMI), adjusted femoral neck hip BMD, adjusted lumbar spine BMD, and adjusted total hip BMD in different genotypes in the studied subjects are reported in Table 3. Our data indicated that the* VDR* p.Gly14Ala genetic variant was statistically associated with adjusted femoral neck hip BMD, adjusted lumbar spine BMD, and adjusted total hip BMD. Individuals with genotype GG showed significantly higher adjusted BMD value than those of genotypes GC and CC (*P* values < 0.05, Table 3). As for p.His305Gln, significant differences within adjusted femoral neck hip BMD, adjusted lumbar spine BMD, and adjusted total hip BMD were detected among different genotypes in the studied subjects. Individuals with genotype CC had significantly higher adjusted BMD value than those of genotypes CG and GG (*P* values < 0.05, Table 3).

## 4. Discussion

It has been confirmed that the osteoporosis is a common skeletal disorder characterized by low bone density caused by decreased bone turnover and increased activity of osteoclasts [1–9, 13, 49]. Growing evidence supports that the* VDR* gene is one of the most important candidate genes in the modification of BMD metabolism and the pathogenesis of osteoporosis. Genetic variations in the* VDR* gene have been proved to be potential associated with BMD and osteoporosis [8–32]. However, results from these published studies were conflicting but not conclusive. The mechanism underlying the association of* VDR* genetic variations with BMD and osteoporosis still remains uncertain. In the present study, we evaluated the genetic influence of the p.Gly14Ala and p.His305Gln genetic variants of* VDR* gene with the susceptibility to BMD and osteoporosis in Chinese postmenopausal women. Our data indicate that there are significant differences in the distribution of allelic and genotypic among primary postmenopausal osteoporosis patients and healthy controls for the p.Gly14Ala and p.His305Gln genetic variants of* VDR* gene (*P* values < 0.05, Table 2), and these two genetic variants were statistically associated with the decreased susceptibility to BMD and osteoporosis (*P* values < 0.05, Table 2). Therefore, results from this study suggest that the allele-C and genotype CC of p.Gly14Ala and the allele-G and genotype GG of p.His305Gln genetic variants in* VDR* gene could contribute to the risk of BMD decrease in Chinese postmenopausal women (Table 3). The* VDR* p.Gly14Ala and p.His305Gln c.269C>A genetic variants are nonsynonymous coding mutations that caused amino acid replacement in* VDR *protein. These two genetic variants might be linked to other* VDR* genetic variants (such as Fok I (rs10735810), BsmI (rs1544410), and ApaI (rs7975232)) which have been proved to affect the function of* VDR* protein and contribute to influence the development of BMD and osteoporosis. Results from those published reports could support our findings. Our data provide more evidence to support that the* VDR* genetic variants could influence the development of BMD and osteoporosis. Therefore, we hypothesize that the p.Gly14Ala and p.His305Gln genetic variants of* VDR* gene play similar functions in the development of BMD and osteoporosis. Our findings indicate that the* VDR* p.Gly14Ala and p.His305Gln genetic variants were statistically associated with adjusted femoral neck hip BMD, adjusted lumbar spine BMD, and adjusted total hip BMD. Results from this study suggest that the* VDR* p.Gly14Ala and p.His305Gln genetic variants are significantly associated with BMD and osteoporosis in Chinese postmenopausal women and might be used as molecular markers for assessing the risk of osteoporosis.

## 5. Conclusions

In conclusion, this case-control study is first to report the influence of* VDR* p.Gly14Ala and p.His305Gln genetic variants on BMD and osteoporosis in the Chinese postmenopausal women. Our findings indicate that these two genetic variants may contribute to influence the pathogenesis of BMD and osteoporosis. More epidemiological and mechanistic studies with large different ethnic populations are necessary to confirm our findings and to further evaluate the role of* VDR* genetic variants in regulating BMD and osteoporosis in the future.

## Figures and Tables

**Table 1:** The PCR amplification and CRS-PCR analysis for investing VDR genetic variants.

SNPs	Primer sequences	PCR amplification fragment size (bp)	Annealing temperature (°C)	Region	Restriction enzyme	Genotype (bp)
p.Gly14Ala	5′-CACTTCCCTGCCTGACCCTA-3′ 5′-GTGAAAAATGCAAGGGCTCC-3′	213	59.8	Exon3	*Mae*I	GG: 195, 18 GC: 213, 195, 18 CC: 213

p.His305Gln	5′-ATTGTCTCTCACAGCCGGTCA-3′ 5′-GGCCCTCAGCAGGTCTTTGT-3′	206	61.4	Exon9	*Mae*III	CC: 189, 17 CG: 206, 189, 17 GG: 206

Note: VDR: vitamin D receptor; SNPs: single nucleotide polymorphisms; PCR: polymerase chain reaction; CRS-PCR: created restriction site-PCR. Underlined nucleotides mark nucleotide mismatches enabling the use of the selected restriction enzymes for discriminating sequence variations.

**Table 2:** The allelic and genotypic frequencies of *VDR* genetic variants in the studied subjects.

Groups	p.Gly14Ala							p.His305Gln						
	Genotypic frequencies (%)			Allelic frequencies (%)		*χ* ^2^	*P*	Genotypic frequencies (%)			Allelic frequencies (%)		*χ* ^2^	*P*
	GG	GC	CC	G	C			CC	CG	GG	C	G		
Case group (*n* = 482)	210 (43.57)	198 (41.08)	74 (15.35)	618 (64.11)	346 (35.89)	5.5553	0.0622	222 (46.06)	207 (42.94)	53 (11.00)	651 (67.53)	313 (32.47)	0.2062	0.9020
Control group (*n* = 488)	230 (47.13)	220 (45.08)	38 (7.79)	680 (69.67)	296 (30.33)	2.1758	0.3369	265 (54.30)	176 (36.07)	47 (9.63)	706 (72.34)	270 (27.66)	4.7690	0.0921

Total (*n* = 970)	440 (45.36)	418 (43.09)	112 (11.55)	1298 (66.91)	642 (33.09)	0.7006	0.7045	487 (50.21)	383 (39.48)	100 (10.31)	1357 (69.95)	583 (30.05)	3.5873	0.1664
	*χ* ^2^ = 13.6018, *P* = 0.0011			*χ* ^2^ = 6.7816, *P* = 0.0092				*χ* ^2^ = 6.6290, *P* = 0.0364			*χ* ^2^ = 5.3267, *P* = 0.0210			

**Table 3:** The characteristics of *VDR* genetic variants in the studied subjects.

SNPs	p.Gly14Ala				p.His305Gln			
Genotype	GG	GC	CC	*P*	CC	CG	GG	*P*
Number (%)	440 (0.4536)	418 (0.4309)	112 (0.1155)	—	487 (0.5021)	383 (0.3948)	100 (0.1031)	—
Age (years)	62.2 ± 7.2	62.5 ± 7.3	62.9 ± 6.9	0.553	61.9 ± 7.8	62.8 ± 7.0	62.9 ± 7.5	0.462
Height (cm)	161 ± 7.8	163 ± 6.8	164 ± 6.9	0.438	158 ± 7.8	162 ± 6.2	164 ± 6.9	0.538
Weight (kg)	62.4 ± 6.3	62.6 ± 6.5	62.8 ± 6.1	0.329	61.9 ± 7.7	62.7 ± 6.5	62.9 ± 6.5	0.365
BMI	23.2 ± 3.28	23.4 ± 3.35	23.7 ± 3.42	0.452	23.3 ± 3.67	23.6 ± 3.41	23.7 ± 3.53	0.239
Lumbar spine BMD (g/cm^2^)	0.932 ± 0.117	0.827 ± 0.122	0.822 ± 0.119	0.018	0.925 ± 0.113	0.832 ± 0.120	0.828 ± 0.126	0.028
Femoral neck hip BMD (g/cm^2^)	0.751 ± 0.125	0.687 ± 0.119	0.679 ± 0.128	0.037	0.749 ± 0.202	0.681 ± 0.197	0.679 ± 0.211	0.027
Total hip BMD (g/cm^2^)	0.865 ± 0.134	0.817 ± 0.156	0.809 ± 0.129	0.028	0.861 ± 0.129	0.820 ± 0.219	0.813 ± 0.172	0.048

Note: VDR: vitamin D receptor; SNPs: single nucleotide polymorphisms; BMD: bone mineral density; BMI: body mass index; Data are shown as mean ± SD (BMD values adjusted by age, weight, and height).
